# Karyotype differentiation *in three* species of *Tripogandra* Raf. (Commelinaceae) with different ploidy levels

**DOI:** 10.1590/S1415-47572010005000085

**Published:** 2010-12-01

**Authors:** André Marques, Fernando Roa, Marcelo Guerra

**Affiliations:** Laboratório de Citogenética Vegetal, Departamento de Botânica, Universidade Federal de Pernambuco, Recife, PEBrazil

**Keywords:** *Tripogandra*, *cytotaxonomy*, rDNA sites, CMA/DAPI bands, meiosis

## Abstract

Most species of the genus *Tripogandra* (Commelinaceae) are taxonomically poorly circumscribed, in spite of having a relatively stable basic number *x* = 8. Aiming to estimate the cytological variation among *Tripogandra* species carrying this base number, several structural karyotypic characters were investigated in the diploid *T. glandulosa*, the hexaploid *T. serrulata*, and the octoploid *T. diuretica*. A careful evaluation of chromosome size and morphology did not reveal clear chromosome homeologies among karyotypes. The mean chromosome size was strongly reduced in the octoploid species, but not in the hexaploid species. They also differed largely in the CMA^+^ banding pattern and in the number of 5S and 45S rDNA sites per monoploid chromosome complement. All three species showed proximal DAPI ^+^ heterochromatin, although in *T. serrulata* this kind of heterochromatin was only visible after FISH. Further, the meiosis in *T. serrulata* was highly irregular, suggesting that this species has a hybrid origin. The data indicate that, in spite of the conservation of the base number, these species are karyologically quite different from each other.

The genus *Tripogandra* Raf. (Commelinaceae, tribe Tradescantieae, subtribe Tradescantiinae) comprises approximately 22 species, some of which are restricted to Mexico, while others are distributed throughout Mexico, Central America, and South America (Handlos, 1975). These species are characterized principally by the presence of dimorphic stamens and double cincinni (Handlos, 1975). Recently, molecular data have revealed that some *Tripogandra* species are grouped into one of the clades of the genus *Callisia* (Bergamo, 2003; Evans *et al.*, 2003; Burns, 2006), suggesting that *Tripogandra* is a polyphyletic group. Cytotaxonomic analyses, mainly those that seek to identify the basic chromosome number, have contributed to the understanding of the phylogenetic relationships of many species groups (reviewed by Guerra, 2008), and can be useful in evaluating the relationships among species of this genus.

Within the family Commelinaceae, the subtribe Tradescantiinae, which includes *Tripogandra*, *Callisia*, *Tradescantia*, and *Gibasis*, stands out by its wide variation in basic numbers, chromosome morphology, and chromosome sizes (Jones and Kenton, 1984; Pitrez *et al.*, 2001). The haploid number *n* = 8 and its euploid multiples predominate in *Tripogandra,* with *x* = 8 largely accepted as the basic number of the genus, while *n* = 5, 6, and 8 are encountered in *Gibasis*, *n* = 6, 7, and 8 in *Callisia*, and *n* = 6 or multiples prevail in *Tradescantia* (Jones and Jopling, 1972). Although *Tripogandra*, *Callisia*, *Gibasis*, and *Tradescantia* share the basic number *x* = 8, these genera show great diversity in chromosome morphology and ploidy levels, hindering an understanding of the cytotaxonomy of the subtribe (Handlos, 1970).

Cytogenetic analyses using other karyotype parameters, such as C-banding and staining with fluorochromes, have also contributed to an understanding of the relationships among some Commelinaceae species (Jones and Kenton, 1984; Kenton, 1991; Sakurai and Ichikawa, 2001). Parokonny *et al.* (1992) showed that some cryptic species of *Gibasis* can be clearly distinguished using techniques such as fluorescence *in situ* hybridization (FISH) and genomic *in situ* hybridization (GISH). However, this type of chromosome analysis is still restricted to just a few genera of the subtribe, and the cytology of a number of genera, such as *Callisia* and *Tripogandra,* has not been studied yet with any of these techniques.

In the present work several structural karyotype characteristics were analyzed in three species of *Tripogandra* (*T.diuretica*, *T. glandulosa,* and *T. serrulata*) with *x* = 8, in order to understand the cytotaxonomical relationships among them. We also attempted to identify which structural characteristic of these karyotypes was best conserved and could represent a karyological synapomorphy of the genus. We investigated the structure of the interphase nuclei, the distribution of heterochromatin by fluorochrome staining with chromomycin A_3_ (CMA) and 4',6-diamidino-2-phenylindole (DAPI), and the number and location of the 5S and 45S rDNA sites by *in situ* hybridization (FISH). Previous chromosome counts had reported 2*n* = 16 for *T. glandulosa*, 2*n* = 32, 39, 48, ca. 50 for *T. serrulata*, and 2*n* = 62 + 1B, 64 for *T. diuretica* (Simmonds, 1954; Celarier, 1955; Lewis *et al.*, 1967; Handlos, 1970; Jones and Jopling, 1972; Pitrez *et al.*, 2001). The meiotic behavior of the two polyploid species was also analyzed in order to evaluate the stability of these species.

## Materials and Methods

###  Plant material

Samples of *Tripogandra diuretica*, *T. glandulosa*, and *T. serrulata* were analyzed. All plant samples investigated grew in the experimental garden at the Department of Botany of the Federal University of Pernambuco, and the vouchers were deposited in the UFP herbarium of this university. Voucher numbers, provenances, and general cytological data for each sample are presented in Table 1.

###  Slide preparation

Root tips were pre-treated with 8-hydroxyquinoline (0.002 M) for 5 h at 18 °C, fixed in absolute alcohol/glacial acetic acid (3:1, v/v) for 2-24 h at room temperature, and stored at -20 °C. Subsequently, they were washed three times in distilled water and digested in a 2% (w/v - 0.02 U/mL) cellulase (Onozuka) and 20% (v/v - 120 U/mL) pectinase (Sigma) solution for 1-2 h at 37 °C. Meristems were squashed in a drop of 45% acetic acid and frozen in liquid nitrogen to remove the coverslip. The slides were stained with DAPI (2 μg/mL):glycerol (1:1, v/v) solution to permit selection of the best cells. Subsequently, the slides were destained in ethanol/glacial acetic acid (3:1, v/v) for 30 min at room temperature and transferred to absolute ethanol overnight at 10 °C. Slides were air dried and aged for three days at room temperature for CMA/DAPI staining (Barros-e-Silva and Guerra, 2010).

For meiotic analysis, young inflorescences from *T. diuretica* and *T. serrulata* were fixed and stored as previously described. Prior to slide preparation, the material was washed twice in distilled water, anthers were squashed in a drop of 45% acetic acid, and the coverslip was removed after freezing in liquid nitrogen.

###  CMA/DAPI staining

For CMA/DAPI fluorochrome banding, aged slides were stained with 0.1 mg/mL CMA (Sigma) for 1 h and counterstained with 1 μg/mL DAPI (Sigma) for 30 min. The slides were mounted in 1:1 (v/v) McIlvaine's buffer-glycerol with 2.5 mM MgCl_2_ (pH 7.0) and kept in the dark at room temperature for 3 days before analysis, as described by Barros-e-Silva and Guerra (2010). Well-spread cells were captured with a CCD Cohu camera on a Leica DMLB epifluorescence microscope using the Leica Q-FISH software. The slides were destained again and stored at -20 °C.

###  Fluorescence *in situ* hybridization (FISH)

FISH procedure was based on Pedrosa *et al.* (2002) with minor modifications. Briefly, slides were treated with RNase at 37 °C for 1 h, washed three times, in 2x SSC for 5 min each , and digested with pepsin at 37 °C for 20 min. The preparations were post-fixed in 1% (para)formaldehyde in 1x PBS for 10 min and dehydrated in an ethanol series.

Two rDNA probes, D2 and R2, were used to locate the 5S and 45S rDNA sites, respectively. D2 is a 500-bp fragment of the 5S rDNA repeat unit from *Lotus japonicus* (Pedrosa *et al.*, 2002) and R2 is a 6.5-kb fragment that contains a 45S rDNA repeat unit from *Arabidopsis thaliana* (Wanzenböck *et al.*, 1997). The D2 probe was labeled with Cy3-dUTP (Invitrogen) and the R2 probe was labeled with digoxigenin-11-dUTP (Life Technologies) by nick translation. The hybridization mixture contained 50% formamide (v/v), 10% dextran sulfate (w/v), 2x SSC, and 2-5 ng/μL of probe. The probe mixture was denatured at 75 °C for 10 min and the chromosome denaturation was undertaken at 75 °C for 6 min. After overnight hybridization at 37 °C, slides were washed in the following order: 2x SSC at room temperature, 2x SSC, 0.1x SSC, and 2x SSC at 42 °C, and 2x SSC at room temperature, for 5 min each. The digoxigenin probe was detected with sheep anti-digoxigenin FITC conjugate (Dako) and amplified with rabbit anti-sheep FITC conjugate (Roche). All slides were mounted in DAPI (4 μg/mL) that was diluted 1:1 (v/v) in Vectashield (Vector). Metaphase images were acquired as indicated above and were later edited in Adobe Photoshop CS3.

###  Chromosome measurements and idiograms

The total chromosome length (S) and the chromosomes arm ratios (AR = long/short arm) were estimated using the Adobe Photoshop CS3 software. An idiogram summarizing most karyotype data for each species was constructed based on chromosome measurements of four well-spread cells, disregarding the length of the secondary constriction. The chromosomes were ordered by size from the largest to the smallest.

## Results

###  Chromosome numbers, chromosome morphology, and nuclear structure

The karyotype parameters of the *Tripogandra* species analyzed are shown in Table 1 including average chromosome length.

**Figure 1:**
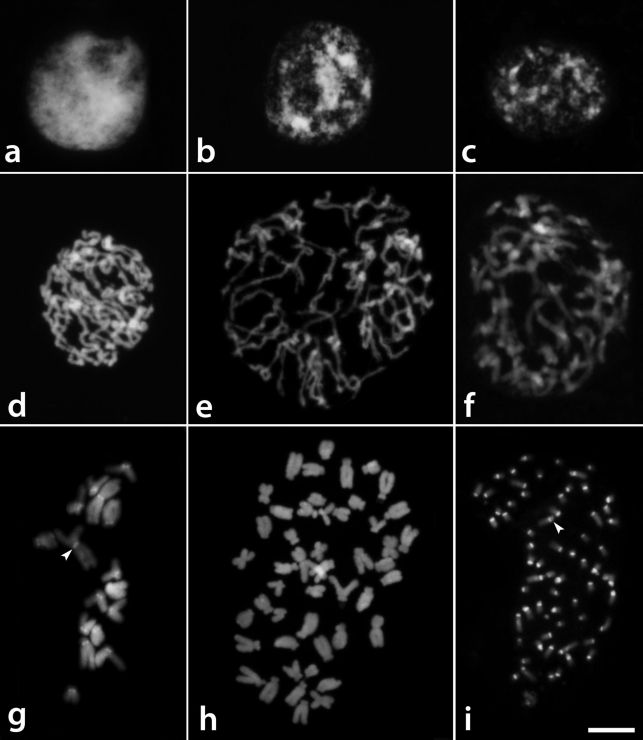
DAPI stained cells of *Tripogandra* species showing the interphase nuclear structure (a-c), the prophase chromosome condensation patterns (d-f), and the metaphase chromosomes (g-i). a, d, g - *T. glandulosa*; b, e, h - *T. serrulata*; c, f, i - *T. diuretica*. Observe the variations in chromatin density and distribution in the interphase nuclei and in the chromosomes at prophase. Arrowheads point to centromeric bands in g, i. Bar corresponds to 10 μm.

According to the nomenclature that was proposed by Delay (1949) for interphase nuclei, *T. glandulosa* exhibited reticulate nuclei, with a dense and uniformly distributed chromatin structure, *T. diuretica* nuclei showed a semi-reticulate structure with irregularly distributed chromatin, and *T. serrulata* nuclei displayed an intermediary chromatin structure between these two types (Figures 1a-c). Condensation of the prophase chromosomes was uniform along the chromosomes of *T. glandulosa* (Figure 1d), while there were some regions that were less condensed than others in *T. diuretica* and *T. serrulata* (Figures 1e,f).

###  CMA/DAPI staining and FISH

**Figure 2:**
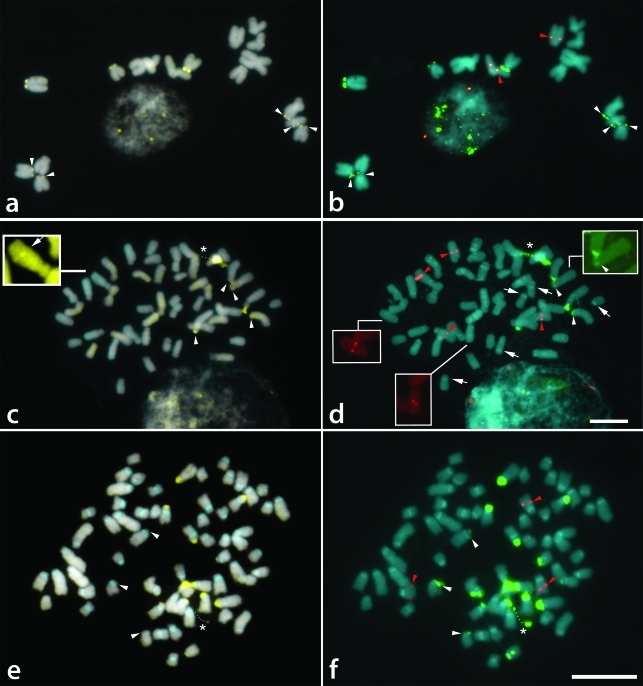
Distribution of CMA/DAPI bands (a, c, e) and rDNA sites (b, d, f) in *Tripogandra* species. a, b - *Tripogandra glandulosa*; c, d - *T. serrulata*; e, f - *T. diuretica.* White arrowheads indicate small CMA^+^ bands and small 45S rDNA sites. Red arrowheads indicate 5S rDNA sites. Arrows in d point to DAPI^+^ bands that appear only after FISH. Dots in c-f indicate secondary constrictions (asterisks). Insert in c shows CMA^+^ band co-localized with 5S rDNA; inserts in d show weak 5S (red) and 45S rDNA (green) signals. Bar in d is valid for a-d and in f is valid for e-f. Bars correspond to 10 μm.

The pericentromeric regions of all chromosomes of *Tripogandra glandulosa* (2*n* = 16) were generally observed as weak DAPI^+^ bands, which were better contrasted after FISH (Figure 1g). CMA^+^ bands were observed on the short arms of the eight acrocentric chromosomes (Figure 2a), with a pair of them showing a much larger and brighter band. The distribution of the 45S rDNA sites coincided with the eight CMA^+^ bands, while the 5S rDNA sites were localized in the interstitial/proximal regions of the long arms of two acrocentric chromosomes (Figure 2b).

The CMA^+^ bands of *T. serrulata* (2*n* = 48) were also co-localized with 45S rDNA sites on the short arms of six acrocentric chromosomes, four of which were brighter than the other two (Figure 2c,d). DAPI^+^ bands were not observed with direct CMA/DAPI staining (Figure 1h), although weak heterochromatic bands were sometimes observed after FISH in the interstitial and centromeric regions (not included in the idiograms) (Figure 2d). 5S rDNA sites were located in the interstitial region of three pairs of acrocentric chromosomes, one of them with very small signals (inserts in Figure 2d). Exceptionally, one of these sites was found co-localized with a CMA^+^ band (see insert in Figure 2c).

**Figure 3:**
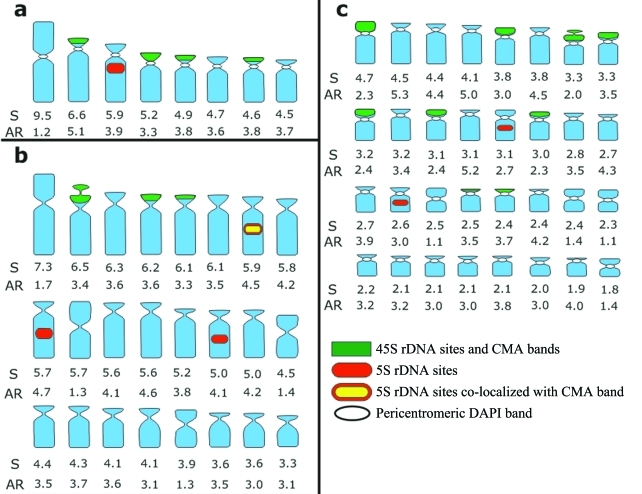
Idiograms of the three species of *Tripogandra* analyzed, including chromosome size in μm (S), arm ratio (AR), distribution of rDNA sites, and CMA and DAPI bands. a - *Tripogandra glandulosa* (2*n* = 16); b - *T. serrulata* (2*n* = 48); c - *T. diuretica* (2*n* = 64).

*Tripogandra diuretica* (2*n* = 64) had DAPI^+^ bands in the pericentromeric region of all chromosomes (Figure 1i), and CMA^+^ bands co-localized with 45S rDNA sites on the short arms of 10 submetacentric and eight acrocentric chromosomes, three of them weakly stained (arrowheads in Figures 2e,f). In some cells, three chromosome pairs with 45S rDNA sites appeared to have short arms slightly longer due to the decondensation of the nucleolar organizing regions. We observed 5S rDNA sites in the interstitial region of a pair of submetacentric chromosomes, and also in one small acrocentric chromosome (Figure 2f). The idiograms in Figure 3 summarize these data. Chromosome pairs homozygous or heterozygous for bands or rDNA sites were represented in the idiograms as a single chromosome having these marks.

###  Meiotic analysis

**Figure 4:**
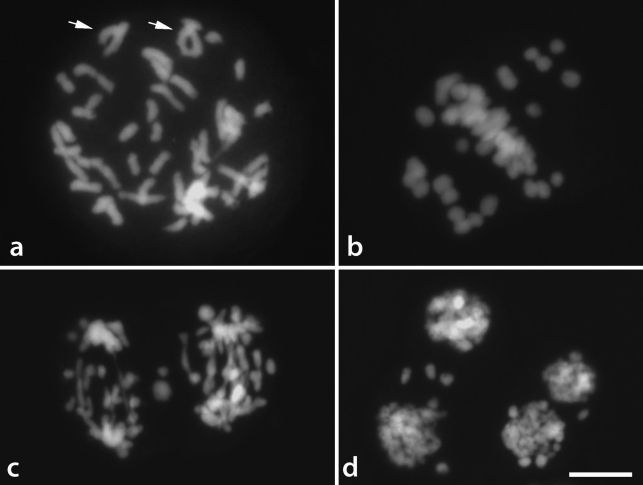
Irregular meiosis in *Tripogandra serrulata.* a – Diakinesis, arrows point to bivalents with one or two (ring) chiasmas. b – Metaphase I with ca.15 bivalents and ca.18 univalents. c – Anaphase II with several bridges and laggard chromosomes; d – Telophase II with micronuclei. Bar corresponds to 10 μm.

Meiosis in *T. serrulata* was irregular, with the formation of many univalents and only a few bivalent chromosomes in most diakinesis and metaphase I cells analyzed (Figures 4a,b). In the remaining cells the pairing configuration was not clear. Bridges and laggard chromosomes were observed in most cells in anaphase I and II, and one to seven micronuclei were found in telophase II (Figures 4c,d). *Tripogandra diuretica*, on the other hand, showed regular meiosis with the formation of 32 bivalent chromosomes in metaphase I and micronuclei in only 3% of the tetrads.

## Discussion

The three species of *Tripogandra* analyzed here had distinct karyotypes and different ploidy levels (2*x*, 6*x*, 8*x*). In addition to the chromosome number 2*n* = 48 (hexaploid) that was commonly observed in *T. serrulata* (Celarier, 1955; Lewis *et al.*, 1967; and the present work), Handlos (1970) reported some tetraploid individuals with 2*n* = 32. The chromosome numbers 2*n* = 39 and 2*n* = ca. 50 for *T. serrulata* reported by Lewis *et al.* (1967) and Simmonds (1954), respectively, are probably miscounts.

The karyotype formulae of the three species analyzed here [*T. glandulosa*, 2*n* = 16 (2M + 14A); *T.serrulata*, 2*n* = 48 (6M + 2SM + 40A); *T. diuretica*, 2*n* = 64 (8M + 12SM + 44A)] showed a number of metacentric chromosomes that were proportional to those expected based on the karyotype formulae of the diploid *T. glandulosa*. However, the metacentric chromosomes of the hexaploid *T.serrulata* and the octoploid *T. diuretica* were much smaller than those observed in *T. glandulosa*, suggesting that they may be non-homeologous. The divergence is even more evident in the number of acrocentric chromosomes expected (56A) and observed (44A) in the octoploid. Therefore, these polyploids are probably not directly related to *T. glandulosa*.

The average haploid set length (HSL) of *T. glandulosa* complement (45.6 μm) and *T. serrulata* (123.8 μm) was proportional to the ploidy level and DNA amount (*T. glandulosa*, 1C = 3.2 pg; *T. serrulata*, 1C = 9.75 pg; Bennett and Leitch, 1995). On the other hand, the HSL of *T. diuretica* was much smaller than expected, based on the variation on ploidy level, a trend to genome downsizing more commonly found in old polyploids (Leitch and Bennett, 2004**).** No DNA content estimate was found for this species**.** The difference in the average chromosome size of *T. serrulata* and *T. diuretica* suggests that these polyploids represent different evolutionary lineages, with *T. serrulata* being younger than *T. diuretica*. Accordingly, these species differ in several morphological characters, such as the number of stamens, style/ovary relative length, type of stigma, fruit form, and surface of seed testa (Handlos, 1970, 1975). In addition, *T. diuretica* has a South American distribution from Bolivia to Argentina, whereas *T. serrulata* occurs from Mexico to Peru and the Caribbean.

The two polyploids also differed in their meiotic behavior. *Tripogandra diuretica* showed a completely regular meiosis, whereas *T. serrulata* displayed a series of meiotic irregularities, including an elevated number of univalents, anaphase bridges, and micronuclei. Curiously, Celarier (1955) reported a hexaploid specimen of *T. serrulata* with 24 bivalents and normal meiosis, suggesting the existence of different hexaploid cytotypes within this species. Thus, *T. serrulata* seems to be a complex species, having populations with normal meiosis as well as populations with highly irregular meiosis.

Analyses of the interphase nuclear structure of the three species examined here revealed an additional difference in their chromatin organization, which was reticulate in the diploid, semi-reticulate with chromocenters in the octoploid, and intermediate between these types in the hexaploid. This variation depends in part on the average chromosome size, which is generally larger in species with reticulate nuclei (Guerra, 1987). Interestingly, the differences in the interphase nuclei were not reflected in the condensation pattern of their prophase chromosomes, as has been observed in other species (Delay, 1949; Guerra, 1987).

The heterochromatin in these species is of two distinct types: DAPI^+^, observed in the proximal regions of *T. glandulosa* and *T. diuretica*, and CMA^+^, which was terminally located in all three species. The DAPI^+^ bands observed in *T. serrulata* only after FISH are probably not AT-rich and not similar to the DAPI^+^ bands of the other two species (Barros-e-Silva and Guerra, 2010). The CMA^+^ heterochromatin was always co-localized with 45S rDNA sites, except in a chromosome of *T. serrulata* in which it was co-localized with a 5S rDNA site. In general, only the 45S rDNA sites co-localize with CMA^+^ bands (Moraes *et al.*, 2007; Souza *et al.*, 2009), although in some species the CMA^+^ bands have been found co-localized with both 5S and 45S rDNA sites (Cabral *et al.*, 2006). The number of CMA^+^ bands and 45S rDNA sites varied broadly among the three species, without correlation to ploidy level, pointing once again to the instability of such chromosome landmarks (see Pedrosa-Harand *et al.*, 2006; Kovarik *et al.*, 2008).

The different karyotype parameters analyzed here varied among the three species, indicating that the basic number is the only cytological feature clearly shared by all three species. In general, *T. glandulosa* and *T. serrulata* were more similar to each other than to *T. diuretica* in chromosome sizes, karyotype formulae, and number of 5S rDNA sites per monoploid chromosome complement.

## Figures and Tables

**Table 1:** Karyotype parameters of the *Tripogandra* species analyzed, with respective voucher numbers and provenance.

\=tbody Species	2*n*	Ploidy level	Average chromosome length (range)^*a*^	HSL*^a^	Karyotype formula^*b*^	Heterochromatin and rDNA sites^*c*^	Voucher-Herbarium	Provenance
*Tripogandra glandulosa* (Seub.) Rohweder	16	2x	5.7 (4.5-9.2)	45.6	2M + 14A	16p DAPI^+^ + 8t CMA^+^/45S rDNA + 2p 5S rDNA	44635-UFP	Posadas, Argentina
*T. serrulata* (Vahl) Handlos	48	6x	5.2 (3.3-7.3)	123.8	6M + 2SM + 40A	6t CMA^+^/45S rDNA + 6i 5S rDNA	44634-UFP	French Guiana^*d*^
*T. diuretica* (Mart.) Handlos	64	8x	2.9 (1.8-4.7)	92.2	8M + 12SM + 44A	64p DAPI^+^ + 18t CMA^+^/45S rDNA + 3i 5S rDNA	44383-UFP	Rio de Janeiro, Brazil

^*^HSL, Haploid set length; ^*a*^values in micrometers; ^*b*^M, metacentric; SM, submetacentric; A, acrocentric; ^*c*^i, interstitial; p, proximal; t, terminal; ^*d*^unknown municipality.
